# Alterations in RNA editing in skeletal muscle following exercise training in individuals with Parkinson’s disease

**DOI:** 10.1371/journal.pone.0287078

**Published:** 2023-12-22

**Authors:** Heather Milliken Mercer, Aiswarya Mukundan Nair, Angela Ridgel, Helen Piontkivska

**Affiliations:** 1 Department of Biological Sciences, Kent State University, Kent, OH, United States of America; 2 Department of Biological and Environmental Sciences, University of Mount Union, Alliance, OH, United States of America; 3 School of Health Sciences, Kent State University, Kent, OH, United States of America; 4 Brain Health Research Institute, Kent State University, Kent, OH, United States of America; 5 Healthy Communities Research Institute, Kent State University, Kent, OH, United States of America; Fondazione Don Carlo Gnocchi, ITALY

## Abstract

Parkinson’s Disease (PD) is the second most common neurodegenerative disease behind Alzheimer’s Disease, currently affecting more than 10 million people worldwide and 1.5 times more males than females. The progression of PD results in the loss of function due to neurodegeneration and neuroinflammation. The etiology of PD is multifactorial, including both genetic and environmental origins. Here we explored changes in RNA editing, specifically editing through the actions of the Adenosine Deaminases Acting on RNA (ADARs), in the progression of PD. Analysis of ADAR editing of skeletal muscle transcriptomes from PD patients and controls, including those that engaged in a rehabilitative exercise training program revealed significant differences in ADAR editing patterns based on age, disease status, and following rehabilitative exercise. Further, deleterious editing events in protein coding regions were identified in multiple genes with known associations to PD pathogenesis. Our findings of differential ADAR editing complement findings of changes in transcriptional networks identified by a recent study and offer insights into dynamic ADAR editing changes associated with PD pathogenesis.

## Introduction

Parkinson’s Disease (PD) is the most common motor neurodegenerative disease with disability and death due to PD increasing faster than any other neurological disorder [1]. Prevalence of PD is 1% over the age of 60 but increases to approximately 4% over the age of 80 [2, 3]. The characteristic symptoms of PD including resting tremor, bradykinesia, rigidity, and postural instability [3–5] are largely due to the loss of dopaminergic neurons in the substantia nigra pars compacta [3]. A specific hallmark of PD is the accumulation of protein, particularly those composed of alpha-synuclein, called Lewy bodies, however, these aggregations are not common across all cases of PD [3, 6]. While PD disease mechanisms, heterogeneity of PD symptoms, and the loss of dopaminergic neurons is not fully understood [3], it is recognized that PD tends to aggregate in families [7–10] and that DNA sequence variants play a role in disease development [3]. Several genetic mutations associated with some PD cases have been identified such as autosomal dominant mutations in SNCA [11], LRRK2 [12], and VPS35 [13, 14] and autosomal recessive mutations in Parkin [15], DJ-1 [16], PINK1 [17], and DNAJC6 [18]. Several other genes have been associated with parkinsonism, which characterizes the motor symptoms associated with PD, with many more genes identified as putative PD genes [3].

While the effects of PD within the central nervous system (CNS) are well documented [3, 19–21], the resulting impacts on skeletal muscle are not fully understood [22]. Kelly et al. [23] observed a pathological phenotype in the skeletal muscle of PD patients characterized by the abnormal grouping of type I myofibers when compared to changes that occur in normal aging. Type I fibers, or slow oxidative fibers, contract more slowly than other types of myofibers, produce lower powered contractions, and are resistant to fatigue making them useful in maintaining posture and stabilizing bones and joints. The degree of type I grouping in PD patients is associated with signature motor recruitment patterns [23, 24] and is used as a measure to assess symptom severity on the Unified Parkinson’s Disease Rating Score (UPDRS). The abnormal groupings, presumed to result from the denervation-reinnervation process, indicate that impaired neuromuscular junction integrity, associated with excessive motor unit activation during weight-bearing activities, may be at play in PD etiology [25].

Several studies have shown that high-intensity resistance training increases muscle strength and improves symptoms in individuals with PD [23, 26–30]. The expression of 704 unique genes was altered in skeletal muscle of PD patients following high-intensity resistance training in a recent study. The changes in expression correlated with increases in muscle mass and strength, improved cognition, feelings of well-being, scores on the (UPDRS) and the Parkinson’s Disease Questionnaire (PDQ-39) [22]. Further, the decreased neuronal signaling observed in the brains of PD patients was positively improved following exercise as evidenced by the increase in the amplitude of low-frequency fluctuations within the right ventromedial prefrontal cortex (PFC), left ventrolateral PFC, and bilaterally within the substantia nigra (SN) [31]. These data show that exercise not only affects the cellular biology within the skeletal muscle of PD patients, but also initiates changes in the CNS.

While many studies have focused on differential gene expression in the progression of PD [32–36], other genetic influences may factor into the neuromuscular manifestation and pathogenesis of PD. RNA editing by Adenosine Deaminases Acting on RNA (ADARs), which causes adenosine to inosine deamination, is the most common mechanism of post-transcriptional RNA editing in the nervous system and integral to neurodevelopment [37–40]. Unlike gene mutations, which result in permanent changes to the genome, RNA editing can be dynamically and differentially regulated and controlled spatiotemporally in a nuanced manner [41, 42]. Patterns of ADAR editing vary among species in terms of editing levels, targets, and ADAR isoforms, with the vast majority of editing occurring in non-coding regions of the genome [43–45]. Two mammalian proteins, ADAR1 (ADAR) and ADAR2 (ADARB1), are catalytically active and expressed in many tissues. In particular, ADAR1 has an interferon-inducible isoform, underlying a connection between the immune system activation and RNA editing [46]. Both ADAR1 isoforms can be found in the nucleus or cytoplasm however the p150 isoform tends to accumulate in the cytoplasm due to its strong nuclear export signal encoded within its sequence [47]. ADAR2 is generally localized to the nucleus [47] and sequestered within the nucleolus leading to an overall decrease in the editing of ADAR2 targets [48, 49]. Studies of alternative splicing of ADAR2 pre-mRNA have suggested that as many as 9 ADAR2 isoforms may exist with varying substrate specificity [50, 51]. ADAR3 (ADARB2) does not show catalytic activity and is only found in the brain, however it is thought to be important during neurodevelopment as an editing inhibitor [40]. While post-transcriptional RNA editing is widespread, its role in skeletal muscle is not completely understood. Hsieh et al. [52] and Noda et al. [53] suggest that RNA editing is integral to the process of myogenesis although other studies have inferred that the editing level is relatively low in skeletal muscle compared to other types of tissue [54, 55]. These seemingly contradictory findings suggest potential dynamic relationships between RNA editing events and editing patterns in myogenesis.

Although lower in numbers, RNA editing sites in protein coding regions and miRNAs are relatively conserved in mammals and can lead to recoding events [45, 56–60]. Notably, recoding events can have profound phenotypic consequences due to a mere single nucleotide change within a key amino acid encoding codon. ADAR editing has been implicated in the development and progression of multiple neurological, neurodegenerative and psychiatric disorders such as epilepsy [61–64], autism [65, 66], schizophrenia [67], Amyotrophic Lateral Sclerosis (ALS) [68–71], Huntington’s Disease (HD) [67], Alzheimer’s Disease (AD) [67, 72–74], schizophrenia [75–80], suicide [75, 77, 78, 80], and depression [81, 82]. Similar to other neurodegenerative diseases, PD is a complicated disease with multiple phenotypes that vary across individuals, as well as by age and sex [83, 84]. Therefore, it is likely that multiple complicating factors such as the aging process, hormones, genetic and epigenetic factors, including dynamic ADAR editing, may play a role in PD pathology and/or response to exercise.

Utilizing a list of 737 genes [85] known to function in PD pathology and a list of 704 unique genes shown to be differentially expressed in Pre-Training PD and Post-Training PD samples [22] (S1 File), we examine whether ADAR editing patterns change due to age and disease state within proteins whose function has been linked with the manifestation and progression of PD. We further compared editing patterns between pre- and post-training PD skeletal muscle samples, to determine whether rehabilitative exercise facilitates changes in ADAR editing. Our results show shifts in ADAR editing patterns between groups and offer insights into dynamic gene regulatory mechanisms underlying PD pathogenesis and response to exercise.

## Materials and methods

### Transcriptomics dataset

The transcriptomics dataset used in this study included samples from various subgroups from the skeletal muscle RNA-seq study of Lavin et al. [22], namely, samples representing Older Males category (n = 9), PD Males (n = 9), Younger Males (n = 9), and Post-training PD Males (n = 4), respectively. Category designations followed Lavin et al. [22] definitions, with Older Males ranged from 50–79 years old, PD Males from 54–76 years old, Younger Males from 23–35 years old, and Post-Training PD Males from 54–76 years old. Pre-training PD sample data (n = 4) was included in the PD Male category, when making comparisons to Older Males, Younger Males, and Post-Training PD Males but were also used independently for within-patient comparisons to Post-Training PD samples. Used independently, the Pre-training PD vs Post-Training PD data comparison provides an investigation of editing changes in PD within the individual following exercise training as these samples came from the same subjects. Post-Training participants completed a 16-week resistance rehabilitative training program, as described in Lavin et al. [22]. Briefly, this program consisted of thrice weekly sessions of strength, power, endurance, balance, and functional training lasting between 35–45 minutes. Because the Lavin et al. [22] study included only a handful of female samples (Older Females = 3, PD Females = 3, Younger Females = 3, and Post-Training Females = 1), and our prior studies have suggested sex-specific differences in ADAR editing patterns [86], we focused our analyses on male samples. Nonetheless, the results of a preliminary analysis on this small subset are discussed briefly in our results. Table 1 lists the pairwise comparisons between analyzed groups.

**Table 1:** Pairwise comparisons between control and treatment groups, and changes in ADAR editing parameters if any.

Pairwise comparison between groups	Differences attributed to	Mean number of ADAR editing events (+/-SEM) per sample for each group
Older Males (n = 9) vs PD Males (n = 9)	Effects of PD	6284.1 ± 200.8 vs 6468.2 ± 130.3
Older Males (n = 9) vs Younger Males (n = 9)	Effects of aging	6284.1 ± 200.8 vs 5902.6 ± 355.4
PD Males (n = 9) vs Post-Training PD males (n = 4)	Effects of exercise	6468.2 ± 130.3 vs 6477.8 ± 680
Pre-Training Males (n = 4) and Post-Training Males (n = 4)	Effects of exercise	6360.5 ± 133.2 vs 6477.8 ± 680

* Pre- and post-training comparison is a smaller subset of a larger PD Males to Post-Training PD males comparison; however, the former subset only includes PD patients that have both pre- and post-training samples, for stricter within-patient analysis. ** None of the pairwise comparisons reached the level of statistical significance using Mann-Whitney test (p value >0.05). Values for each group are given as mean and SEM, standard error of mean.

### ADAR editing inferences from RNA-seq data

RNA-seq data from Lavin et al. [22] study was downloaded from NCBI SRA (Bioproject PRJNA588234; GSE140089) [87]. Samples were sequenced using paired 50-bp-long reads, with the number of mapped reads varying between $\tilde{3}3.7$ million (33,726,174) and $\tilde{8}2$ million (82,025,036), with an average of $\tilde{5}9.9$ million (59,956,071) reads per sample. Information on mapped reads of samples utilized in this study are shown in S2 File.

The Automated Isoform Diversity Detector (AIDD) pipeline [88] was used to infer ADAR editing events. Briefly, fastq files of individual patients were trimmed and aligned to the chosen human reference (GRCh37) using HISAT2 [89]. Once aligned, the transcriptome was assembled using StringTie [90], to estimate levels of individual gene expression as Transcripts Per Kilobase Million (TPM). ADAR editing events were inferred using GATK haplotype caller [91] following the best practices, as described in Plonski et al. [88]. VCF files are available at https://doi.org/10.5281/zenodo.7971793. SnpEff [92] was used to predicts the effects and functional impacts of predicted edited variants, as described below. Variants identified are putative ADAR editing events described as such based on known ADAR editing sites, but for simplicity will be further described throughout this study as simply “editing events”.

### Inferences of editing consequences

A list of 758 genes with suspected associations to PD were downloaded from NCBI Gene (downloaded April 20, 2022) [85] and converted to Ensembl gene and transcript IDs, resulting in 737 gene and 7827 transcript IDs (S1 File) utilizing Ensembl Biomart [93]. The NCBI PD Gene List (n = 737) is one of the multiple GeneRIF (Gene Relevance into Function) lists, which are created and frequently updated to reflect current research through three primary methods: extraction from published literature by the National Library of Medicine staff, summary reports from HuGE Navigator [94], and user submissions from a Gene record [95]. Additionally, we utilized a list of 302 genes observed to be upregulated in skeletal muscle of PD patients following exercise and 402 genes observed to be downregulated following exercise in a recent study by Lavin et al. [22] resulting in a list of 704 unique genes which will be referred to as Lavin Differentially Expressed (DE) gene list throughout this study. Both gene lists can be found in S1 File.

We used the DAVID interface [96–98] to identify Reactome [99] pathways in which PD and Lavin DE genes were over-represented including only pathways with at least 10 genes, and at least 10% of genes in the sample gene list associated with the pathway, as well as reported FDR p-value less than 0.05. For consistency, we also included pathways where some categories did not meet the “10% of a pathway” criteria if that was met in at least one other category.

SnpEff [92] annotations were collated by sample and patient group. NCBI PD genes found within PD, control, and Post-training PD samples, were compared between groups and analyzed for gene overrepresentation within Reactome pathways [99] using the Database for Annotation, Visualization, and Integrated Discovery (DAVID) [96–98] interface. A similar analysis was performed following the same procedure focusing on edits associated with genes found in the Lavin DE list. We focused on Reactome pathways because a larger number of our genes of interest (75.8% for PD gene list and 75.9% for Lavin DE gene list) had assigned Reactome annotations, compared to KEGG pathways annotations (67.5% for PD gene list and 67.9% for Lavin DE gene list). Only genes affected by high or moderate impact editing events occurring in a specific transcript in at least 2 samples were considered similar to procedures followed by other studies [100, 101] in order to reduce stochastic editing events. High impact editing events include those A-to-G or T-to-C nucleotide changes that result in large duplications or deletions, deleted or duplicated exons, frameshifts, gene deletions, etc. Moderate impact editing events include those that result in exon deletion or duplication, codon deletion or insertion, nonsynonymous coding, etc. Significance of overrepresentation is calculated as a p-value with Binomial Test and False Discovery Rate (FDR) using the Benjamini-Hochberg approach [99]. Additionally, we compared the proportion of high and moderate impact editing events occurring in NCBI PD genes and Lavin DE genes, independently, between sample groups using *χ*^2^ test.

We also used SNPNexus [102] interface for SIFT (Sorting Intolerant from Tolerant) [103] analyses of editing effects on protein function and nonsynonymous coding. Only genes and transcripts from the NCBI PD gene list [85] an absence of dbSNP association, and A/G and/or T/C variants were used in this analysis. In an effort to reduce stochastic ADAR editing events and provide increased stringency to our data filtering process, editing events occurring in less than 2 samples were removed similar to procedures outlined by other studies [100, 101]. Similar to SnpEff annotations, the genes in which deleterious and tolerated editing events occurred were further scrutinized to identify Reactome pathways in which those genes were over-enriched.

### Changes in ADAR editing rate

The focus of this study was not specifically on ADAR editing rates, in part due to the small sample sizes available; instead, we focused on those editing events that might have downstream functional and/or regulatory consequences to proteins that may influence PD pathology. Nonetheless, we were able to identify 55 putatively edited sites that were shared across all (male) samples and groups, allowing us to get a preliminary look at differential editing rates between groups. Only edited sites with at least 100 reads were considered. Editing rate was calculated for each site by dividing the total number of edited reads (G reads at an A reference site, or C reads at a T reference site by the total number of aligned reads for that site (i.e., stack depth). These putative editing sites were annotated with a gene/protein Ensembl ID using SNPNexus [102] interface. Results can be found in S5 File and S7 Fig.

### Statistical analyses

*χ*^2^ test was used to compare the numbers of editing events across various categories, such as editing events with high and moderate impact, protein coding, nonsense-mediated decay, deleterious, and tolerated, using GraphPad Prism (version 9.5.1) [104] when categorical data was being analyzed. For ADAR expression, non-parametric Kruskal-Wallis tests were used to compare Older Males, PD Males, Younger Males, Pre-training PD Males, and Post-training PD Males. For comparing the total number of putative ADAR editing events per sample, as a proxy of ADAR editing levels, non-parametric Mann-Whitney test was used.

## Results

We would first like to note that the implications of our findings should be thought of in terms of dynamic downstream changes to gene regulation within functional (such as Reactome) pathways stemming from identified (putative) ADAR editing events within gene members of these pathways, rather than attempting to categorize such changes into groups where “edited” is synonymous with “disease causing” and “unedited” signifies a “non-disease causing” event. Importantly, both directions of editing events should be considered as editing change; in other words, status of an editing change can be assigned whether the transcript that is normally edited but is no longer edited in a specific condition, or if the transcript becomes edited in a specific circumstance. In fact, many genes experience ADAR editing as part of their normal regulation, and thus, aging is expected to correlate with changes in editing patterns [40, 105]. Likewise, some genes stop being edited as part of the normal developmental progression [38] or due to physiological changes [42, 106, 107]. Thus, the major challenge is to understand whether or how shifts in editing patterns across multiple genes within a pathway reflect downstream changes in gene regulation and subsequent physiological changes, including the disease process. Thus, in this study we primarily focus on interpreting observed shifts in ADAR editing events in the context of functional consequences to affected genes, including likely changes in gene regulation patterns among impacted pathways.

### Comparison of overrepresented Reactome pathways between the NCBI PD gene list and the Lavin DE gene list reveal overlapping pathways

We performed Reactome pathway analyses of the Lavin DE gene list (n = 704) and the NCBI PD gene list (n = 737) using the DAVID interface to identify pathways in which each gene list was over-represented, including only pathways with at least 10 genes and at least 10% of genes in each list associated with the pathway as well as reported FDR p-value less than.05. We included pathways where some categories did not meet the “10% of a pathway” criteria if that was met by the other gene list for consistency and comparison purposes. Shared pathways between the two gene lists included Immune System (PD gene list 24.5%, Lavin DE gene list 13.9%), Cytokine Signaling in the Immune System (PD gene list 11.6%, Lavin DE gene list 5.3%), Signal Transduction (PD gene list 27.7%, Lavin DE gene list 20.1%), Generic Transcription Pathway (PD gene list 12.2%, Lavin DE gene list 9.6%), RNA Polymerase II Transcription (PD gene list 12.2%, Lavin DE gene list 10.1%), Gene Expression (Transcription) (PD gene list 13.1%, Lavin DE gene list 11.1%), and Developmental Biology (PD gene list 10.0%, Lavin DE gene list 8.0%). The two gene lists differed in two categories as the PD gene list was overrepresented in two pathways, Innate Immune System (PD gene list 14.2%, Lavin DE gene list 0%) and Disease (PD gene list 17.6%, Lavin PD gene list 0%). On the other hand, the Lavin DE gene list was not found to be over enriched in these pathways, perhaps, reflecting physiological changes indicative of different tissues and disease state and the similarities observed between the two gene lists representing overlapping pathways and gene expression necessary for multiple tissues and cell types. Results of the pathway analysis of the NCBI PD gene list and the Lavin DE gene list can be found in S1 Fig.

The Lavin et al. [22] differentially expressed gene list (n = 704 unique genes) from skeletal muscle samples was compared to the NCBI PD gene list [85] (n = 737), identifying 38 unique genes in common between the two lists, which will be referred to as the Genes of Interest in PD gene list for clarity (S1 File). The 38 Genes of Interest in PD genes were compared to 16,485 Pre-Training PD genes highlighting 32 genes in common, and therefore experiencing editing events. The Genes of Interest in PD were compared to 16,785 Post-Training PD genes identifying 30 genes in common. No significant differences were observed between edited and unedited Genes of Interest in PD genes between Pre- and Post-Training PD samples (p = 0.5540) however the genes being edited in common between the groups and those that are uniquely edited in each group represent genes with known association to PD, have been observed to be differentially expressed between Pre-and Post-Training PD samples [22], and are being differentially edited between the two groups (S3 File).

Thirty-two unique Genes of Interest in PD from Pre-Training PD patients and 30 unique Genes of Interest in PD from Post-Training PD patients all showed over-enrichment in Reactome hierarchical pathways including Immune System, Signal Transduction, Gene Expression, Programmed Cell Death, and Circadian Clock. In Pre-Training PD samples, Cell Response to Stimuli pathway was also enriched. Together, these 88 genes identified either in common between Pre- and Post-Training PD samples or as unique to a group (S3 File) represent a Gene List of Significance that may play a role in the changes in PD symptomatology, ADAR editing, and editing patterns observed between Pre- and Post-Training groups.

### Physiological pathways affected by PD genes in which high or moderate impact editing events are found vary between PD, Control, Pre- and Post-Training PD samples

**Fig 1:**
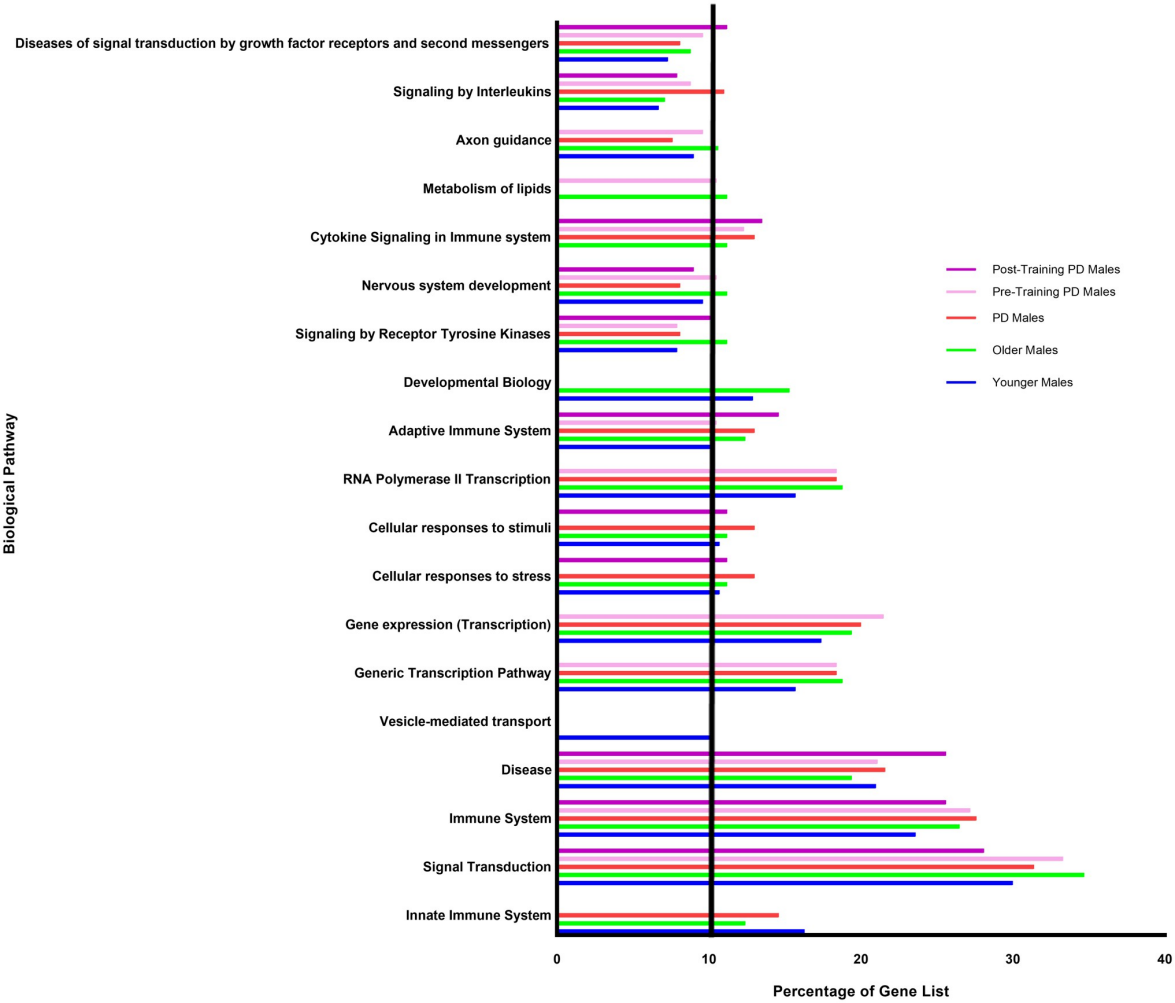
Distribution of ADAR edited genes by overrepresented pathways in each patient category. PD genes in which high or moderate impact editing events occurred within a specific transcript in at least 2 samples within a sample group were analyzed via DAVID to identify overrepresented pathways. Pathways where at least one patient category had at least 10% of genes identified as ADAR edited targets are included. Shifts in editing patterns across pathways can be observed between ages, disease status, and training state.

SnpEff [92] annotations of putative ADAR editing events were filtered for genes in which high or moderate impact editing events occurred within genes from the NCBI PD Gene List. Entries with high or moderate impact editing events in genes that were found in less than two samples were removed to identify 168 unique genes in Older Male samples, 184 unique genes in PD Male samples, 176 unique genes in Younger Male samples, 113 unique genes in Pre-Training PD samples, and 88 unique genes in Post-Training PD Male samples (S3 File). We used the DAVID interface to identify Reactome pathways in which PD genes were over-represented including only pathways with at least 10 genes, and at least 10% of genes in the sample gene list associated with the pathway, as well as reported FDR p-value less than 0.05. For consistency, we also included pathways where some patient categories did not meet the “10% of a pathway” criteria if that was met in at least one other patient category (Fig 1).

Analysis of Reactome pathways in which PD genes were enriched showed that while many pathways were overrepresented among ADAR edited genes across all subject categories such as Signal Transduction, Immune System, Disease, Adaptive Immune System, Signaling by Receptor Tyrosine Kinase, Nervous System Development, Signaling by Interleukins, and Diseases of Signal Transduction by Growth Factor Receptors and Second Messengers. One pathway was uniquely present only in one specific category (Vesicle Mediated Transport in Younger Males) or were enriched for all categories except one including Generic Transcription Pathway, Gene Expression (Transcription), RNA Polymerase II Transcription, and Axon Guidance which were overrepresented in all sample categories except Older Males, Cellular Response to Stress and Cellular Response to Stimuli which were over enriched across all groups except for Pre-Training PD Males, and Cytokine Signaling in the Immune System which was over enriched in all groups except for Younger Males.

When high or moderate impact editing events affecting PD genes are compared between the sample groups, interesting patterns of changes in RNA editing emerge. The proportion of genes showing evidence of high or moderate impact ADAR editing was compared between sample groups to analyze functional relevance (Fig 1). When comparing the ratio of genes devoted to specific functional pathways between Older Males and Younger Males, marked differences were observed in Vesicle Mediated Transport (Older Males 0%, Younger Males 10.1%), Metabolism of Lipids (Older Males 11.2%, Younger Males 0%), and Cytokine Signaling in Immune System (Older Males 11.2%, Younger Males 0%). In Older Males, Developmental Biology was overrepresented while in PD Males, it was not (Older Males 15.3%, PD Males 0%). Metabolism of Lipids pathway also differed between groups (Older Males 11.2%, PD Males 0%). When the pathways in which genes with high or moderate impact edits are over-represented are compared between PD Males and Younger Males, Vesicle Mediated Transport (PD Males 0%, Younger Males 10.1%), Developmental Biology (PD Males 0%, Younger Males 12.9%), and Cytokine Signaling in Immune System (PD Males 13%, Younger Males 0%) varied between the groups. Between Pre-Training PD Males and Post-Training PD Males, differences in pathway over-representation were found between Generic Transcription Pathway (Pre-Training 18.4%, Post-Training 0%), Gene Expression (Pre-Training 21.5%, Post-Training 0%), Cellular Response to Stimuli (Pre-Training 0%, Post-Training 11.2%), Cellular Response to Stress (Pre-Training 0%, Post-Training 11.2%), RNA Polymerase II Transcription (Pre-Training 18.4%, Post-Training 0%), Metabolism of Lipids (Pre-Training 10.5%, Post-Training 0%), and Axon Guidance (Pre-Training 9.6%, Post-Training 0%), but did not differ greatly in the following pathways when the entire PD group was compared to Post-Training samples: Cellular Response to Stimuli (PD Males 13%, Post-Training PD Males 11.2%), Cellular Response to Stress (PD Males 13%, Post-Training PD Males 11.2%), and Metabolism of Lipids (PD Males 0%, Post-Training PD Males 0%). Additionally, when all PD samples are compared to Post-Training PD Males, Innate Immune System pathway is shown to be over-enriched in PD Males, but not in Post-Training PD Males (PD Males 14.6%, Post-Training PD Males 0%).

### The number and proportion of high or moderate impact editing events occurring in PD genes vary between PD, Control, Pre- and Post-Training PD samples

We further explored whether the total number of ADAR editing events observed in PD genes varied between sample groups observing that the increases in the number of ADAR editing events between Younger and Older Males were exacerbated in PD samples, and that the number of editing events decreased when comparing Pre-Training Male samples to the samples collected from the same individuals following exercise. For each subject category, the total editing events included high and moderate impact editing events, along with low impact and modifier editing events. Within Older Male samples, 16,452 total editing events were observed within specific transcripts of PD genes in at least two samples, while there were 18,743 total editing events in PD Males, 16,144 total editing events in Younger Males, 8,408 total editing events in Pre-training PD samples and 6,647 total editing events within Post-training PD samples.

**Fig 2:**
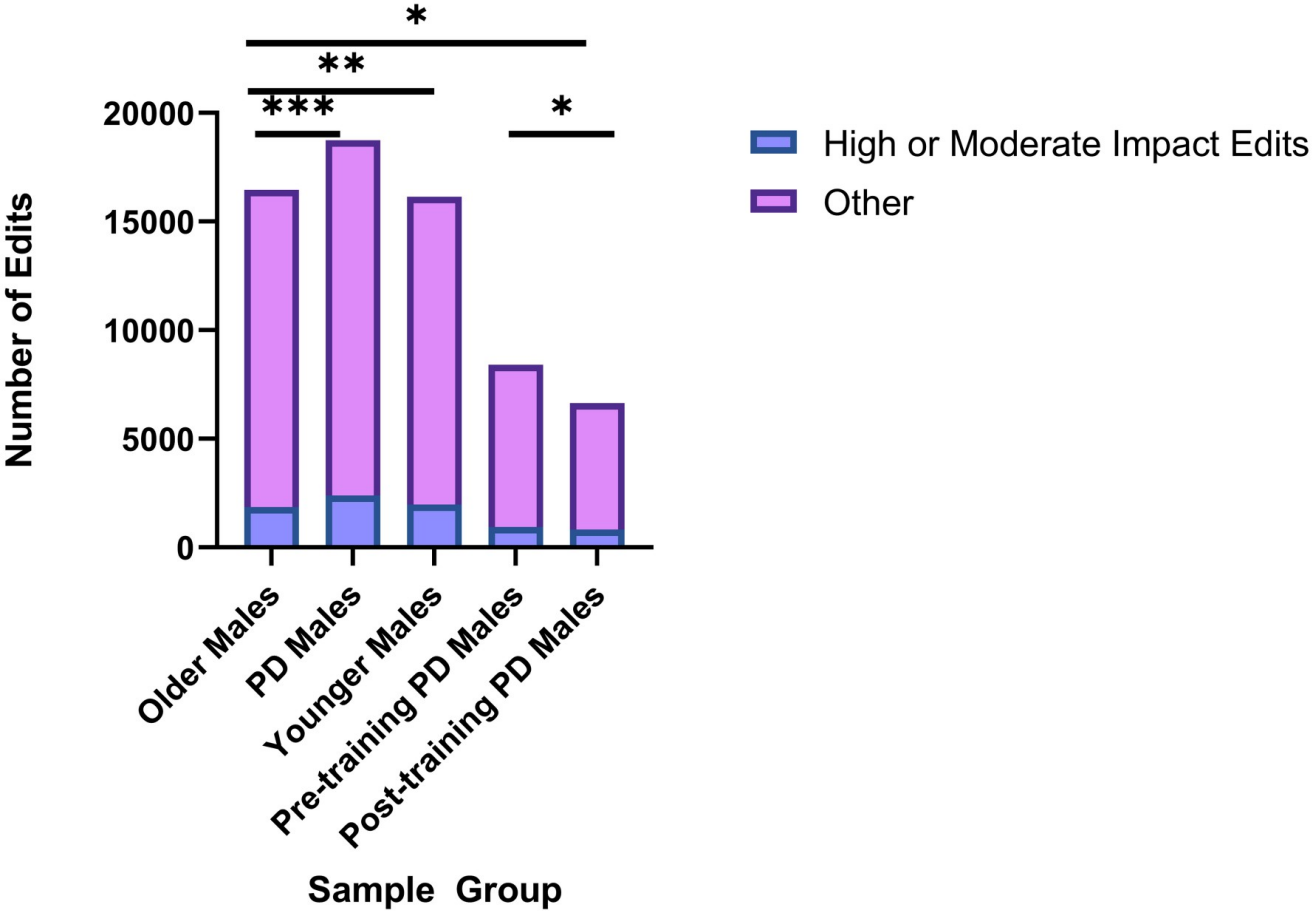
Significant differences in the number of high or moderate editing events in PD genes between sample groups. Significant differences were observed in the number of high or moderate impact editing events in PD genes when comparing editing patterns between Older Males and PD Males (*χ*^2^ test, p = 0.0001), Older Males and Younger Males (p = 0.0065), Pre-Training PD Males and Post-Training PD Males (p = 0.0207), and Older Males and Post-Training PD Males (p = 0.0255). *p = <.05, **p = <.01, ***p = <.001.

Notably, the fractions of high or moderate impact editing events (in other words, those that result in amino acid change with likely functional or structural consequences for affected proteins) also vary between groups with the highest proportions observed in PD Males (13%) although the proportion of high and moderate impact edits decreased when Younger Males were compared to Older Males (12% and 11% respectively). Significant differences in the number of high and moderate impact edits were identified through *χ*^2^ two-tailed analysis when comparing Older Males to PD Males (p = 0.0001), Older Males to Younger Males (p = 0.0065), Pre-training PD Males to Post-Training PD Males (p = 0.0207), and Older Males to Post-Training PD Males (p = 0.0255). Significance was not achieved when comparing the number of high or moderate impact editing events found in PD Males to Younger Male samples (p = 0.1829). Together, the variable proportions of high and moderate impact editing events across sample groups with the editing events occurring in genes associated with different physiological pathways, provides further evidence of dysregulated ADAR editing in PD (Fig 2) (S4 File).

**Fig 3:**
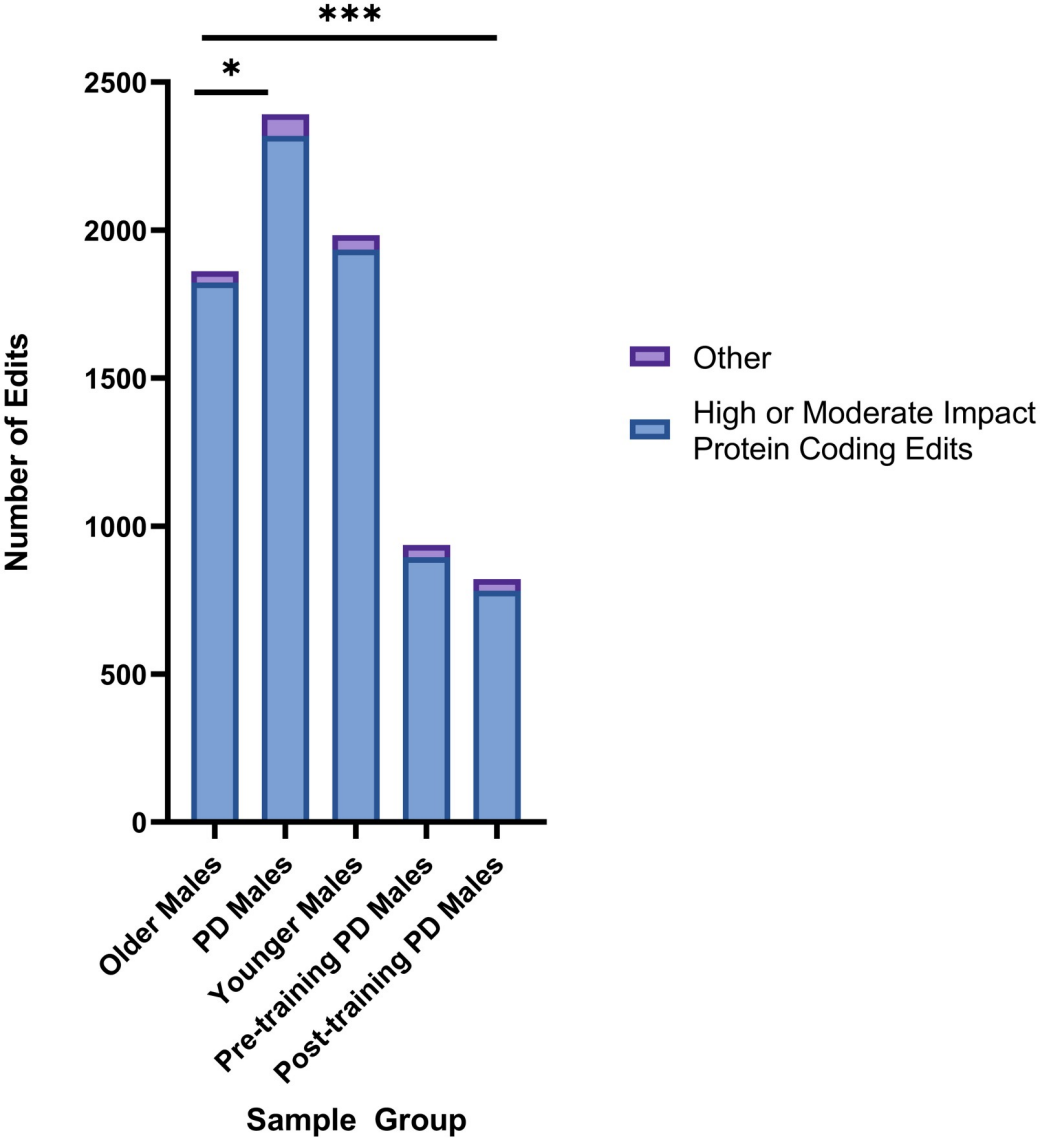
Significant differences in number of high or moderate impact protein coding editing events in PD genes between sample groups. Significant differences were observed in the number of high or moderate impact protein coding editing events when comparing the number of edits between Older Males and PD Males (*χ*^2^ test, p = 0.0482) and Older Males and Post-Training PD Males (p = 0.0001). *p = <.05, **p = <.01, ***p = <.001.

Significance was achieved when comparing high or moderate impact editing events occurring in PD genes in protein coding regions with significant differences identified between Older Males and PD Males (p = 0.0482) and Older Males to Post-Training PD Males (p = 0.0001). Results are shown in Fig 3.

**Fig 4:**
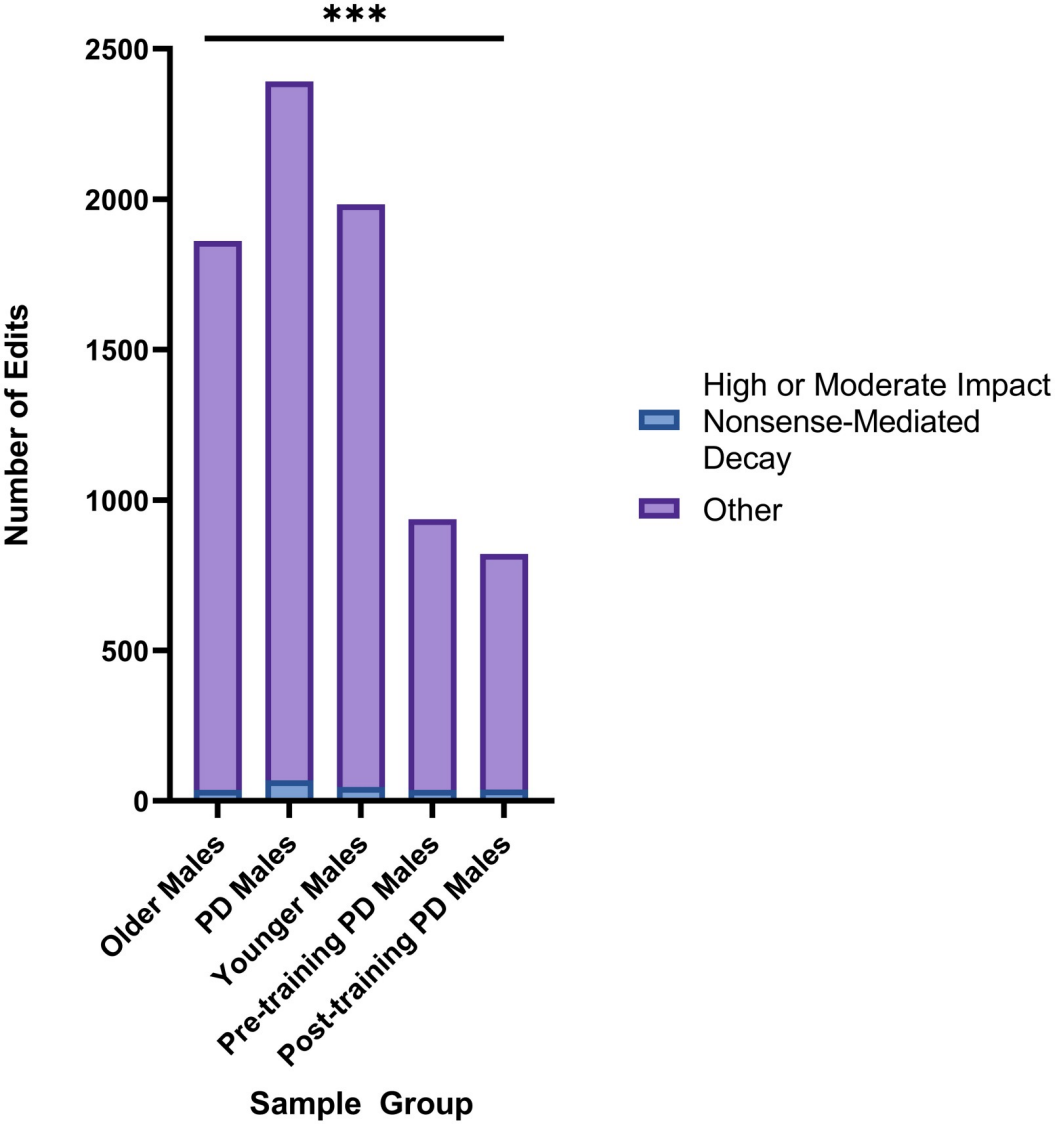
Significant differences in number of high or moderate impact nonsense-mediated decay editing events. Significant differences were observed in the number of high or moderate impact editing events resulting in nonsense mediated decay when comparing the number of edits between Older Males and Post-Training PD Males (Chi-square test, p = 0.0001). *p = <.05, **p = <.01, ***p = <.001.

When we considered the number of high or moderate impact editing events in PD genes that resulted in nonsense-mediated decay, significant differences were identified when comparing Older Males to Post-Training PD Males (p = 0.0001), with the higher fraction of these editing events in Post-Training category (Fig 4). Changes in nonsense-mediated decay regulation have been implicated in a variety of neurodegenerative conditions [108–110], including PD [111], and thus, the observed differences in editing events may point to candidate genes involved in PD pathogenesis and/or those affected by exercise.

**Fig 5:**
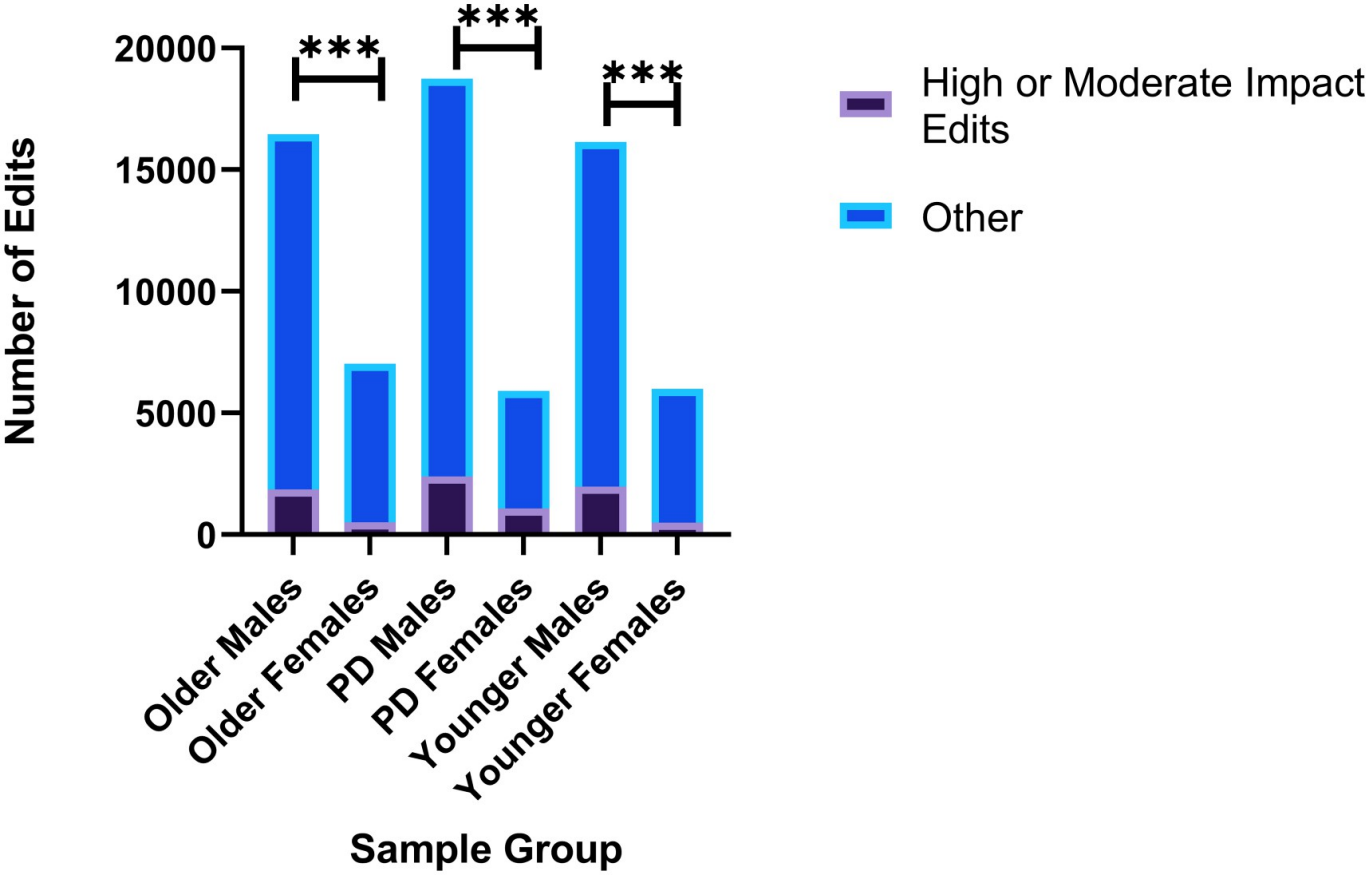
Comparison between the number of high or moderate impact editing events in PD genes between males and females. Significant differences were observed in the number of high or moderate impact editing events when comparing Older Males vs Older Females (*χ*^2^ test, p = 0.0001), PD Males vs. PD Females (p = 0.0001), Younger Males vs. Younger Females (p = 0.0001), and Post-Training PD Males vs one Post-Training PD Female (p = 0.0001). *p = <.05, **p = <.01, ***p = <.001.

The Lavin et al. study [22] also included 10 female samples: 3 Older Females, 3 PD Females, 3 Younger Females, and 1 Post-Training PD Female. This sample number was not robust enough to make valid comparisons between groups and considering our previous findings that ADAR editing may vary between the sexes [86], we are not including an in-depth analysis of female samples along with this research. However, we did conduct a basic analysis of female data, with some intriguing results that we believe warrant future analysis and research. Significant differences in the number of high or moderate impact editing events in PD genes were observed in *χ*^2^ analysis when comparing Older Males to Older Females (p = 0.0001), PD Males to PD Females (p = 0.0001), and Younger Males to Younger Females (p = 0.0001) (Fig 5). We were unable to compare Post-Training PD Males (n = 4) to Post-Training PD Females (n = 1) due to limited available female samples. These pronounced differences suggest that relationships between PD pathology and ADAR editing may be drastically different between the sexes.

### The number and proportion of deleterious and tolerated protein-coding editing events occurring in PD genes vary between PD, Control, Pre- and Post-Training PD samples

**Fig 6:**
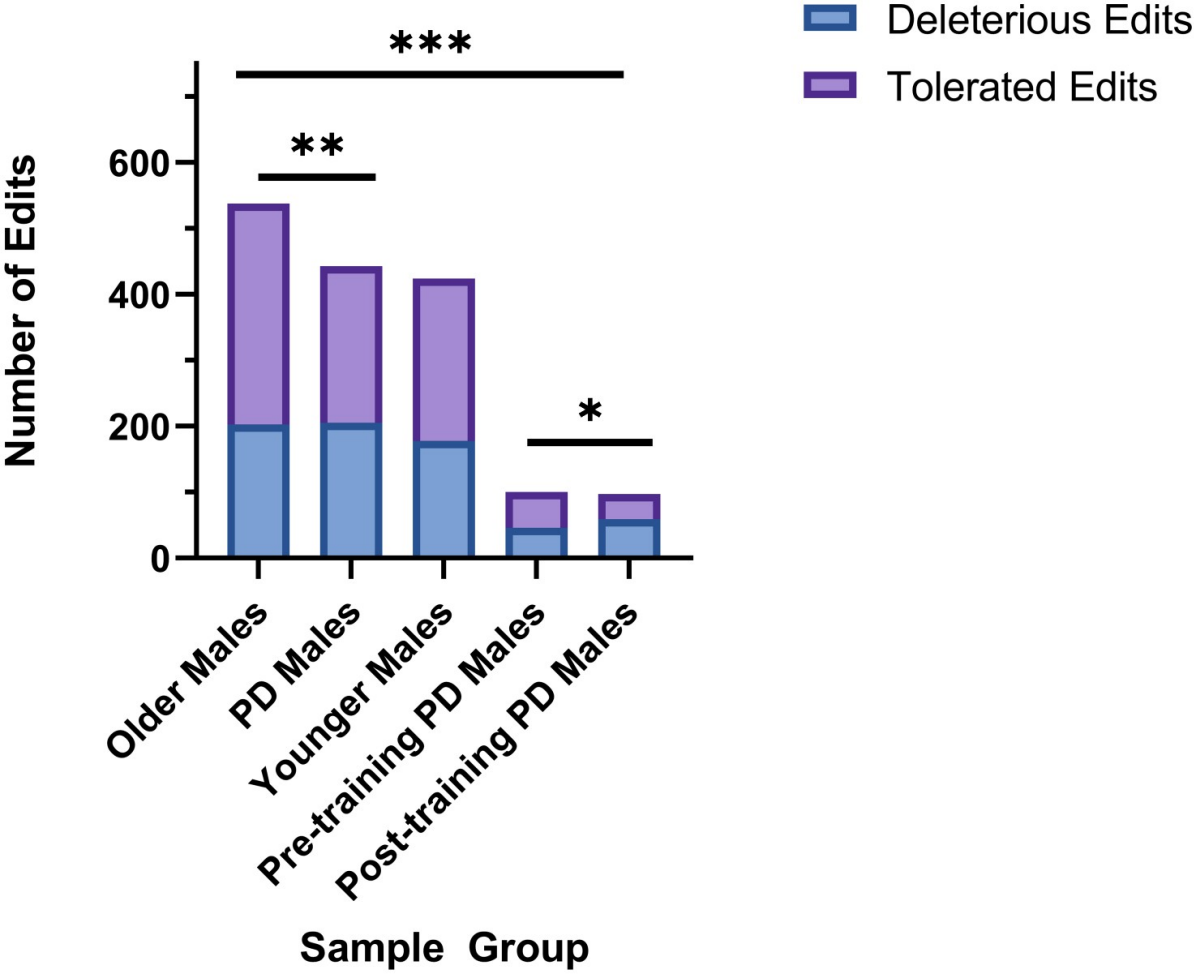
Significant differences in deleterious and tolerated editing events in PD genes on protein outcomes. Significant differences were observed between deleterious and tolerated protein coding editing events as identified by SIFT when comparing Older Males and PD Males (*χ*^2^ test, p = 0.0069), Pre-training PD Males and Post-Training PD Males (p = 0.0371), and Older Males and Post-Training PD Males (p = 0.0001). *p = <.05, **p = <.01, ***p = <.001.

To validate the trends in proportion of high and moderate impact edits in PD genes observed utilizing SnpEff and to further analyze whether these edits result in nonsynonymous coding, we further explored the potential impact of ADAR editing on the proteins from the NCBI PD gene list using SIFT (Sorting Intolerant from Tolerant) [103], through SNPNexus annotations [102]. While SnpEff annotates variants by high impact (implicates loss of protein function), moderate impact (implicates alterations in protein effectiveness), low impact (unlikely to change protein behavior), and modifier (no evidence of impact) and includes a wider range of variant consequences including splice site variations and those that occur in regulatory regions, SIFT categorizes variants as either Deleterious (Damaging to protein function) or Tolerated (unlikely to affect protein function) based on nonsynonymous coding. SIFT assigns a score between 0 and 1 with values less than or equal to.05 being deleterious and greater than.05 designating a tolerated variant. Both high and moderate impact edits as categorized by SnpEff and deleterious edits as designated by SIFT are more likely to result in changes to protein function and/or effectiveness. We only considered deleterious and tolerated A/G and T/C variants with no dbSNP associations that were observed in two or more samples in order to eliminate stochastic edits. *χ*^2^ test was used to analyze the relationship between the amounts of deleterious and tolerated A/G and T/C variants. Significant differences were observed between deleterious and tolerated variants when comparing Older Males to PD Males (p = 0.0069), and Older Males to Post-Training PD Males (p = 0.0001) (Fig 6). Additionally, significance was observed when comparing Pre-training PD Males and Post-Training PD Males (p = 0.0371), which compares individual PD patients before and after exercise training.

### Physiological pathways affected by Lavin DE genes in which high or moderate impact editing events are found vary between PD, Control, Pre- and Post-Training PD samples

SnpEff [92] annotations of putative ADAR editing events were filtered for genes in which high or moderate impact editing events occurred within genes from list of genes found to be differentially expressed in skeletal muscle of PD patients following exercise by Lavin et al. [22]. Entries with high or moderate impact editing events in genes that were found in less than two samples were removed to identify 300 unique genes in Older Male samples, 297 unique genes in PD Male samples, 288 unique genes in Younger Male samples, 174 unique genes in Pre-Training PD samples, and 162 unique genes in Post-Training PD Males. (S4 File) Edited genes were overrepresented across all sample groups in the following Reactome pathways: Gene Expression (Transcription) (Older Males 14.7%, PD Males 15.1%, Younger Males 14.2%, Pre-Training PD Males 17.8%, Post-Training PD Males 14.1%), Signal Transduction (Older Males 21.7%, PD Males 24.2%, Younger Males 24.3%, Pre-Training PD Males 25.3%, Post-Training PD Males 25.2%), RNA Polymerase II Transcription (Older Males 13%, PD Males 13.8%, Younger Males 12.2%, Pre-Training PD Males 16.1%, Post-Training PD Males 12.3%), and Generic Transcription Pathway (Older Males 12%, PD Males 12.8%, Younger Males 11.8%, Pre-Training PD Males 14.9%, Post-Training PD Males 11.7%). Variations in pathway overrepresentation were observed in Developmental Biology and Immune System in which only Older Males and Post-Training PD Males were over enriched for both categories (Developmental Biology: Older Males 10%, PD Males 0%, Younger Males 0%, Pre-Training PD Males 0%, Post-Training PD Males 11%) (Immune System: Older Males 14.7%, PD Males 0%, Younger Males 0%, Pre-Training PD Males 0%, Post-Training PD Males 17.8%), and Adaptive Immune System for which all sample categories were over enriched except for Post-Training PD Males (Older Males 7%, PD Males 9.1%, Younger Males 8%, Pre-Training PD Males 10.3%, Post-Training PD Males 0%) (S2 Fig).

### The number and proportion of high or moderate impact editing events occurring in Lavin DE genes vary between PD, Control, Pre- and Post-Training PD samples

We analyzed the ADAR editing events occurring in the Lavin DE gene list noting, as was observed within PD genes, an increase in the total number of ADAR edits as designated by SnpEff when comparing Younger Males to Older Males, and a further increase when comparing Older Males to PD Males. Additionally, there was a decrease in ADAR edits between Pre and Post-Training samples taken from the same individuals. Total edits included high, moderate, low, and modifier impact edits with 24,291 edits in Lavin DE genes in Older Males, 26,223 edits in PD Males, 22,172 edits in Younger Males, 11,892 edits in Pre-Training PD Males, and 10,356 edits in Post-Training Males. The proportions of high and moderate impact editing events, or those most likely to alter structure and function of affected proteins, also varied between sample groups with significance reached through *χ*^2^ two-tailed analysis when comparing Older Males to Younger Males (p = .0382), PD Males to Post-Training PD Males (p = .0006), and Pre-Training PD Males to Post-Training PD Males (p = .0003). Significance was not reached when comparing Older Males to PD Males (p = .7670) however the trends observed when analyzing high and moderate impact edits in PD genes is also evident when examining Lavin DE gene edits with a general decrease in proportion of high and moderate impact edits from Younger Males to Older Males (7.9% to 7.4% respectively), but not as marked of a difference in proportion from Younger Males to PD Males (7.9% to 7.5%). When examining proportions of high and moderate impact edits in Lavin DE genes between the Pre and Post-Training PD samples from the same individuals, we observe an increase from 7.3% to 8.6% respectively (S3 Fig).

### Expression of ADAR genes vary between disease state, age, and pre-post training samples

**Fig 7:**
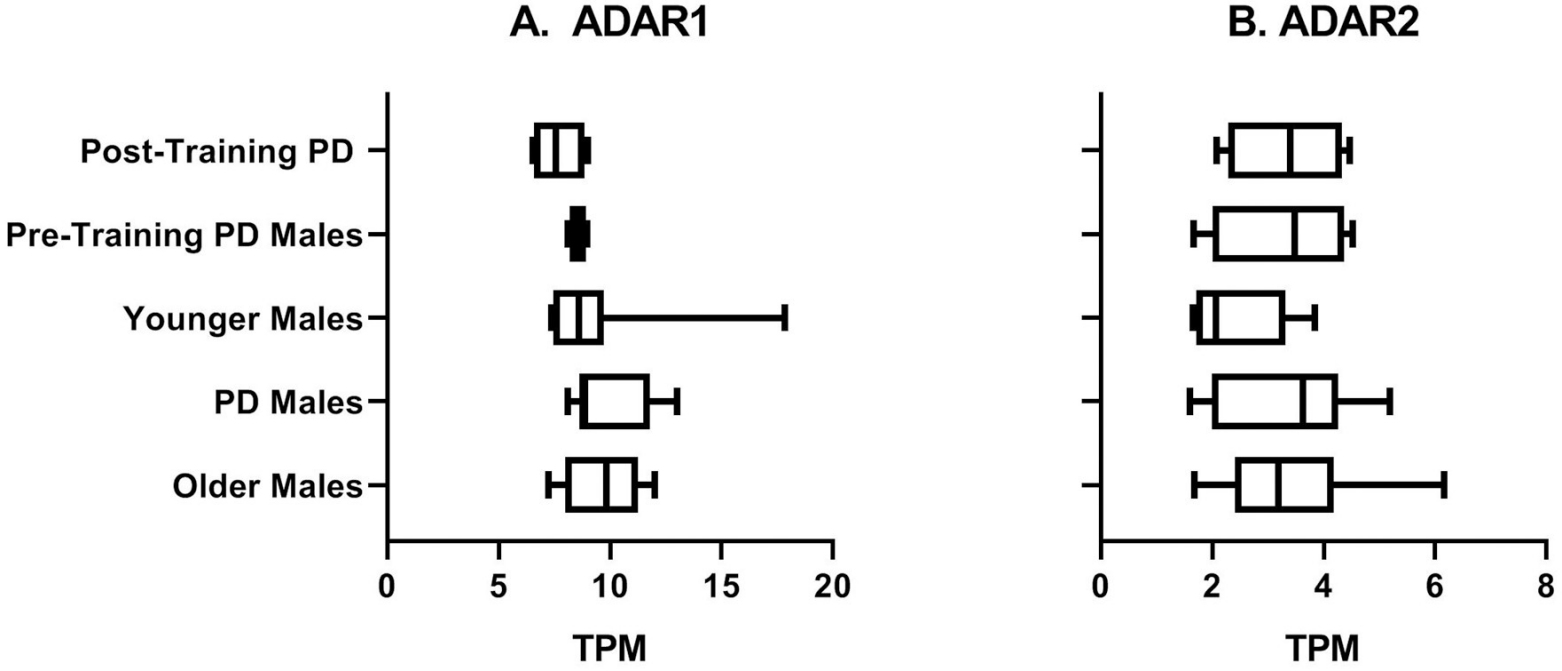
ADAR genes expression levels shown by age, disease state, and training status. (a) ADAR (ADAR1) and (b) ADARB1 (ADAR2) expression, in transcripts per million (TPM), varied somewhat between sample groups, although the pairwise differences did not reach statistical significance (Kruskal-Wallis tests, p values <0.05). ADARB2 (ADAR3) expression (not shown) was minimal in all sample groups as ADARB2 is primarily expressed in the brain.

**Fig 8:**
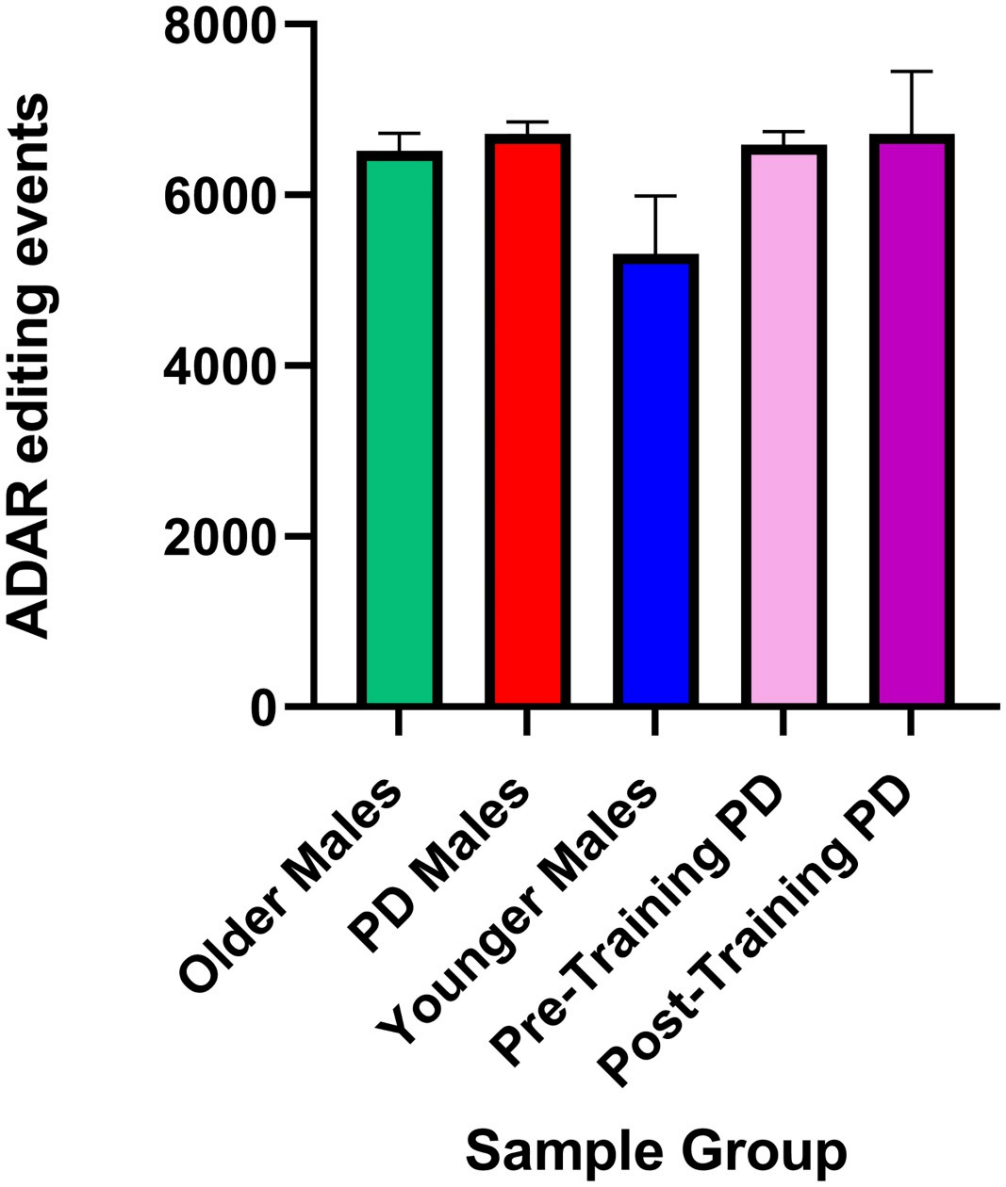
The number of putative ADAR editing events compared between sample groups. Although the mean number of A/G or T/C editing events varied by the group, with the highest number of events identified in PD Males and the lowest number of events observed in Younger Males, none of the pairwise comparisons were statistically significant (Mann-Whitney test, p value <0.05).

While in the Lavin et al. [22] study ADAR genes were not identified as differentially expressed when the entire transcriptomes were considered, because there is a nuanced non-linear relationship between the levels of ADAR editing and expression of individual ADAR genes [47], we wanted to explore whether any differences in ADAR genes expression can be identified among subject groups. ADAR expression was compared between groups revealing marked differences between groups; however, no significance was achieved. The highest expression of ADAR (ADAR1), which includes the interferon-inducible isoform ADARp150 [112], was seen in PD Male samples with 9.83 average TPM followed by Older Males (average TPM = 9.67) and Younger Males (average TPM = 9.45) (Fig 7). The lowest expression of ADAR was demonstrated in Post-training PD Males (average TPM = 7.67) which represented a decrease in ADAR expression when compared to samples from the same individuals prior to exercise training (average TPM = 8.58). The expression of ADARB1 (ADAR2) was similar between Older and PD Males with an average of 3.39 TPM and 3.32 TPM respectively. ADARB1 expression in Younger Males was less than Pre- or Post-Training PD Males with 2.51 TPM in Younger Males, 3.29 TPM in Pre-Training PD Males, and 3.34 TPM in Post-Training PD Males. There was little to no expression of ADARB2 (ADAR3) in any group, as ADARB2 is generally limited to expression in the brain [113]. (Fig 7) The number of ADAR editing events were highest in PD Males (on average, 6,468.2 events per sample) and Post-Training PD Males (6,477.8 events per sample) followed by Pre-Training PD Males (6,360.5 events per sample), Older Males (6,284.1 events per sample) and Younger Males (5,902.6 events per sample) (Fig 8).

### Changes in editing rates reveal specific sites edited differently by ADAR between sample groups

Because the focus of this study is the editing events that might have functional consequences to proteins that may influence PD pathology rather than estimating ADAR editing rates per se, we do not offer an in-depth analysis of differential ADAR editing of our data. Nonetheless, we identified 55 shared sites that were present across male samples, thus, allowing us to consider site to site as well as group to group changes in editing levels (S5 File, S7 Fig). Although the sampling size is not robust enough for definitive conclusions about the effects of aging, PD disease state, or exercise training on ADAR editing in PD, the results provide preliminary evidence that ADAR editing is dysregulated in skeletal muscle in PD, where certain sites have shown lower levels of editing in PD Males compared to Older Males or Younger Males by at least 20%. One of those sites with editing level shifts between groups belongs to actin ACTA1 gene. ACTA1 is not known to be a target of ADAR editing [52], albeit ADAR editing is involved in regulation of myogenesis [114]; thus, this finding needs to be further validated. Interestingly, the editing levels at ACTA1 did not differ as much when Younger Males were compared to Older Males (∼5.6%), or when Pre-Training PD Males were compared Post-Training PD Males (∼1%). Another set of sites of interest for future validation, where PD samples exhibit lower levels of editing compared to either Older Males or Younger Males of at least 20%, belong to a processed pseudogene RP11–750B16.1, implicated in autism spectrum disorder [115].

Other notable sites within protein-coding transcripts with at least 4% editing level changes between PD Males and healthy patients are RPA1 family protein 3 (ARL6IP5), sarcoplasmic/endoplasmic reticulum calcium ATPase 2 (ATP2A2) and phosphoglucomutase PGM1. (S5 File) Noteworthy, the nucleotide change due to ADAR editing would result in a synonymous change in the latter two genes, potentially resulting in changes to mRNA stability [116, 117] and/or translational efficiency [118].

## Discussion

Our results extend Lavin et al. [22] findings of differentially expressed genes to the differences in RNA (ADAR) editing patterns between subject categories, suggesting that exercise training in PD may have effects both on gene expression and differences in RNA/ADAR editing patterns, thus, potentially underlying functionally relevant downstream changes in protein expression and function. In addition, we find that editing patterns change across age groups, and are affected by PD status. From our analysis, we propose the need to further scrutinize the genes in which variations in editing patterns are observed for altered protein function in relation to their editing events and further propose the need for such studies in larger populations and expanded populations that include females.

Specifically, we observed a shift in high and moderate impact editing and affected physiological pathways with potential effects in both genes observed to be important in skeletal muscle [22] and those with known associations to PD [85] when comparing samples by age, disease state, and training status. When comparing the pathways associated with PD genes with high or moderate impact edits between Older and Younger Males, editing within Cytokine Signaling in the Immune System (11.2% of genes in pathway) and Metabolism of Lipids (11.2% of genes in pathway) pathways are over-enriched in Older Males, but not in Younger Males (0% of genes in each pathway), while Vesicle Mediated Transport genes are overrepresented in Younger Males (10.1% of genes in pathway) and not in Older Males (0% of genes in pathway). When the Lavin DE gene list is considered, Developmental Biology and Immune System (10% and 14.7% of genes in pathway, respectively) are over-enriched in Older Males, but not in Younger Male samples (0% of genes in each pathway). When PD genes with high or moderate impact edits found to be overrepresented in Older Males are compared with that of PD Males, they differ in the pathways of Developmental Biology and Metabolism of Lipids with 15.3% and 11.2% of genes represented in their respective pathways and 0% of genes for both categories in PD Males. Developmental Biology genes with high or moderate impact ADAR edits are also over-enriched when Lavin DE genes are considered for Older Males (10% of genes in pathway) vs 0% of genes in the pathway observed in PD Males while 14.7% of genes in the Immune System pathway were observed with high or moderate impact edits in Older Males and 0% of genes in the pathway edited with high or moderate impact in PD Males. Collectively, genes in pathways associated with Developmental Biology, the Immune System, and Metabolism of Lipids pathways were observed to be edited with high or moderate impacts more frequently in Older Males when compared to either PD or Younger Males in this analysis suggesting that this editing pattern may be somehow associated with healthy aging however these trends must be observed in a larger population for these trends to be confirmed.

Although we have limited samples for Pre and Post-Training PD Males, we do observe some interesting patterns when comparing pathway analyses of genes experiencing high and moderate impact editing from samples collected from the same PD patients before and after exercise. When PD genes are considered, General Transcription (18.4% of genes in pathway) and Axon Guidance (9.6% of genes in pathway) pathways are over-enriched in Pre-Training PD Male samples although in Post-Training PD samples, we observe 0% of genes in those pathways experiencing ADAR edits of high or moderate impact. Additionally, we observed 0% of genes in Cellular Response to Stimuli and Cellular Response to Stress pathways edited with high or moderate impact in Pre-Training samples, however in Post-Training samples from the same individuals, we observe 11.2% of genes in both pathways experiencing these types of edits. When editing within Lavin DE genes are assessed, we observe 0% of genes in Developmental Biology and Immune System pathways edited with high or moderate impact in Pre-Training samples, however Post-Training samples show over-enrichment of genes in these pathways with 11% and 17.8% of genes in the pathways experiencing high or moderate impact editing, respectively. Interestingly, we observed that 10.3% of genes in the Adaptive Immune pathway experiencing high or moderate impact editing in Pre-Training samples, but 0% of genes in the pathway edited with high or moderate impact in Post-Training samples suggesting a change in the way that genes functioning in Immune System and Adaptive Immune System pathways are edited in response to exercise. Collectively, we observe a shift in editing patterns between within-patient Pre and Post-Training Male samples although within a small sampling group.

While this study showed that the proportion of high and moderate impact editing events in PD genes decrease with increased healthy aging in this analysis, the trend is not observed when examining the total numbers of putative ADAR editing events. The numbers of A/G and T/C editing events are highest in PD and Post-Training PD samples (on average, 6,468.2 and 6,477.8 editing events, respectively) followed by Pre-Training PD Males and Older Males (6,360.5 and 6,284.1 editing events, respectively), with the lowest number identified among Younger Males (5,902.6 editing events), although none of the pairwise comparisons were statistically significant (Mann-Whitney test, p <0.05) (Table 1). When changes following exercise were considered, the pattern was inconclusive, with half the patients showing 10+% increase in the number of identified editing events Post-Training, and the other half showed the opposite trend. However, this is based on a very small sample of only four patients.

We observe similar patterns when considering edits identified in Lavin DE genes with total number of ADAR edits increasing with increasing age (Younger Males = 22,172 edits, Older Males = 24,291 edits, PD Males = 26,223 edits), and then further elevated in PD Males compared to age-matched healthy samples. On the other hand, we observe a decreased proportion of high and moderate impact edits with increasing age (Younger Males = 7.9%, Older Males = 7.4%, PD Males = 7.5%). Contrary to the PD gene analysis of high and moderate impact editing, we observe a decreased average number of high and moderate impact edits when comparing Pre-Training samples to Post-Training samples (average 2,973 edits per sample and average 2,589 edits per sample respectively), but an increase in proportion between the two groups with 7.3% of edits of high and moderate impact in Pre-Training Males and 8.6% of edits of high or moderate impact in Post-Training PD Males perhaps reflecting the effects of a low sample size. Comparisons of edited genes from the NCBI PD gene list and Lavin DE gene list can be found in S6 and S7 Files, S5 and S6 Figs.

Likewise, ADAR1 shows elevated expression in PD Males (9.83 TPM) followed by Older Males (9.67 TPM) and Younger Males (9.45 TPM). As inflammation levels are known to increase with age [119, 120], it is not surprising that ADAR1 expression is somewhat higher in Older Males than Younger Males; however, we observed even greater elevation in PD. Pozdyshev et al. [121] observed lower expression of ADAR1 and decreased editing levels in PD cases, although their analysis consisted of a larger data set of post-mortem BA9 brain region samples, while our estimates came from muscle biopsies of live patients. Further, we focused on high and moderate impact editing in PD genes and Lavin DE genes rather than overall editing across the genome. Our findings that ADAR editing patterns differ in PD from what may be indicative of healthy aging processes suggests that not only changes in editing enzymes are dynamic between ages and disease, but also that the way that genes are being edited—such as locations and/or level of specific editing events within transcripts—may have downstream significance.

Changes in editing rates were observed between sample groups across a set of specific sites shared among all male samples (S5 File, S7 Fig). Some of those sites (such as ACTA1) show rather stark differences of 20% or more in editing levels between healthy and PD groups. However, because of the relatively small sample sizes and being able to identify only a handful of shared sites due to sequencing depth limitations, these editing changes need to be further validated in larger samples and expanded to broader genome-wide coverage in the future. We further would like to emphasize the need to consider both editing rates and the potential impact of individual edits at both the site- and genome-wide resolutions, as our results show significant variations in high and moderate impact editing as a proxy for differentially edited sites across groups.

A major limitation of this analysis is the absence of subjects’ health histories, medication profiles, and/or knowledge of any co-morbidities that may be present in addition to PD, although the study participants were screened and excluded in the case of specific diagnoses. For example, multiple participants were taking medications related to PD or other pharmaceutical drugs such as cyclooxygenase (COX) inhibitors/NSAIDs [23] which may affect ADAR expression/editing. This medication history may in turn influence whether the extent of editing went up or down post-exercise. However, further studies are necessary to substantiate this effect. As the majority of the samples were procured from individuals of advanced age, the likelihood of comorbidities is elevated [122] and may ultimately affect the functional pathways identified as enriched. The ability to adjust for these factors lies outside the range of this study. In addition, only four subjects had both Pre- and Post-training samples available, thus limiting the power of our analysis. Additional uncertainty can be attributed to unknown differences in sample handling and preparation. Some samples had depth of a less than 50 million reads (S2 File), thus, some editing events may have been missed during variant calling. Moreover, inferences of protein functional outcomes due to nucleotide changes, made by computational tools such as SnpEff and SIFT, may also underestimate the extent of downstream consequences. Lastly, the wide range of PD phenotypes observed in this disease give credence to hypotheses suggesting a broad range of genetic and environmental factors that may contribute to subcategories of PD manifestation [83, 84]; however, the number of samples prevents us from considering PD subcategories.

Importantly, albeit consistent with the bulk of PD research to-date, the lack of female samples creates an obstacle in furthering our understanding of PD, its progression, and how sex impacts its manifestation [22, 86]. While we acknowledge that the extremely small sample size of post-training PD females (n = 1) and other groups makes it difficult to draw conclusions on the effects of exercise training on ADAR editing in PD, we identified intriguing trends that warrant further data collection and research. Many have noted the phenotypic differences in PD between males and females [123–125]. A recent study by Sandor et al. [84] noted gender differences in PD, specifically when considering a specific PD phenotype denoted as ‘Axis 2’ suggesting not only gender influences, but also an interplay between sex and multifactorial genetics. We propose the urgent need to further scrutinize RNA editing patterns in PD, the specific genes involved in significant pathways identified in this study, and the variations in how transcripts are edited between the sexes and among the range of PD phenotypes.

## Conclusion

Here, we have shown that alterations in ADAR editing patterns between PD, Control, and Post-Training PD male samples may lead to changes in protein functions, which although not causal, may contribute to the manifestation and progression of PD pathology. Our findings also show some intriguing changes in ADAR editing patterns that occur post-exercise, although further studies are needed to delineate the role of RNA editing in therapeutic exercise. Likewise, further functional studies are needed for a set of PD-related genes that we identified as harboring high or moderate impact editing events and/or inferred deleterious protein outcomes. Critically, we emphasize the need to further elucidate the role of ADAR editing in PD progression in females, as impact of sex is largely omitted in PD studies to-date.

Ultimately, while mutations in specific candidate genes have been suggested as culprits in PD pathogenesis, no monogenic targets have been definitively identified, making it more likely that a variety of genetic and environmental influences are at play in the manifestation of the neuroinflammation indicative of PD. ADAR editing, a known factor in the pathology of multiple neurodegenerative and psychiatric disorders [46, 88, 106], must be acknowledged as a potential role-player in PD pathology. Further research must strive to understand how dynamic changes in ADAR editing may function as a causal agent in PD progression or whether RNA editing dysregulation is simply an outcome of the neuroinflammation and neurodegeneration present in the disease.

## Supporting information

S1 File Gene lists of NCBI PD genes (downloaded April 20, 2022). Gene Lists of NCBI PD genes (downloaded April 20, 2022), genes up or down-regulated in a study of rehabilitative exercise in PD (Lavin et al., 2020), and genes in common between both lists.
(XLSX)

S2 File Metrics of analyzed RNA-seq data from Bioproject PRJNA588234; GSE140089 (Lavin et al., 2020). (XLSX)

S3 File Ensembl IDs of genes of interest in PD that harbor ADAR editing events in Pre and Post-Training samples in PD. (XLSX)

S4 File Gene lists in which high or moderate impact ADAR edits occur in PD gene and Lavin DE genes, per sample group. (XLSX)

S5 File A comparison of changes in editing rates between sample groups. (XLSX)

S6 File Comparisons of PD genes experiencing high or moderate impact editing between sample groups. (TXT)

S7 File Comparisons of Lavin DE genes experiencing high or moderate impact editing between sample groups. (TXT)

S1 Fig Reactome pathway comparison between NCBI PD genes and Lavin DE gene lists. (TIF)

S2 Fig Reactome pathway analysis of Lavin DE genes experiencing high or moderate impact editing. (TIF)

S3 Fig Significant differences in the number of high or moderate impact editing events in Lavin DE genes between sample groups. (TIF)

S4 Fig Changes in the number of A-to-G and T-to-C editing events PD genes from matched Pre- and Post-Training samples. (TIF)

S5 Fig Comparisons of PD genes experiencing high or moderate impact editing between sample groups. (PNG)

S6 Fig Comparisons of Lavin De genes experiencing high or moderate impact editing between sample groups. (PNG)

S7 Fig Scatterplot of average pairwise editing level differences, per site. Average editing levels are plotted for each pairwise comparison with each dot representing a single site, for all 55 shared sites. The 45-degree line represents the equal levels of editing in two contrasted groups, for a specific site. For all of these comparisons, except between Younger and Older groups, the X axis (baseline) is per the level of editing within PD group (either the entire 9-patients group, or only the subset of 4 patients that participated in exercises). The further away from the 45-degree line the dot is, the larger the difference in editing between the compared groups, for that specific site. As the scatterplots show, there is somewhat more under-edited sites in PD samples compared to either Younger or Older patients; while differences in editing at specific sites is relatively small in Younger vs Older or PD pre- and post-exercise subset of patients. Examples of such under-edited sites include sites from ACTA1 and RP11–750B16.1 (marked).
(TIF)
