# Supplementary figures and images for: Alterations in RNA editing in skeletal muscle following exercise training in individuals with Parkinson’s disease
Source: PLoS One. 2023 Dec 22;18(12):e0287078. doi: 10.1371/journal.pone.0287078 (PMC10745226; doi:10.1371/journal.pone.0287078)

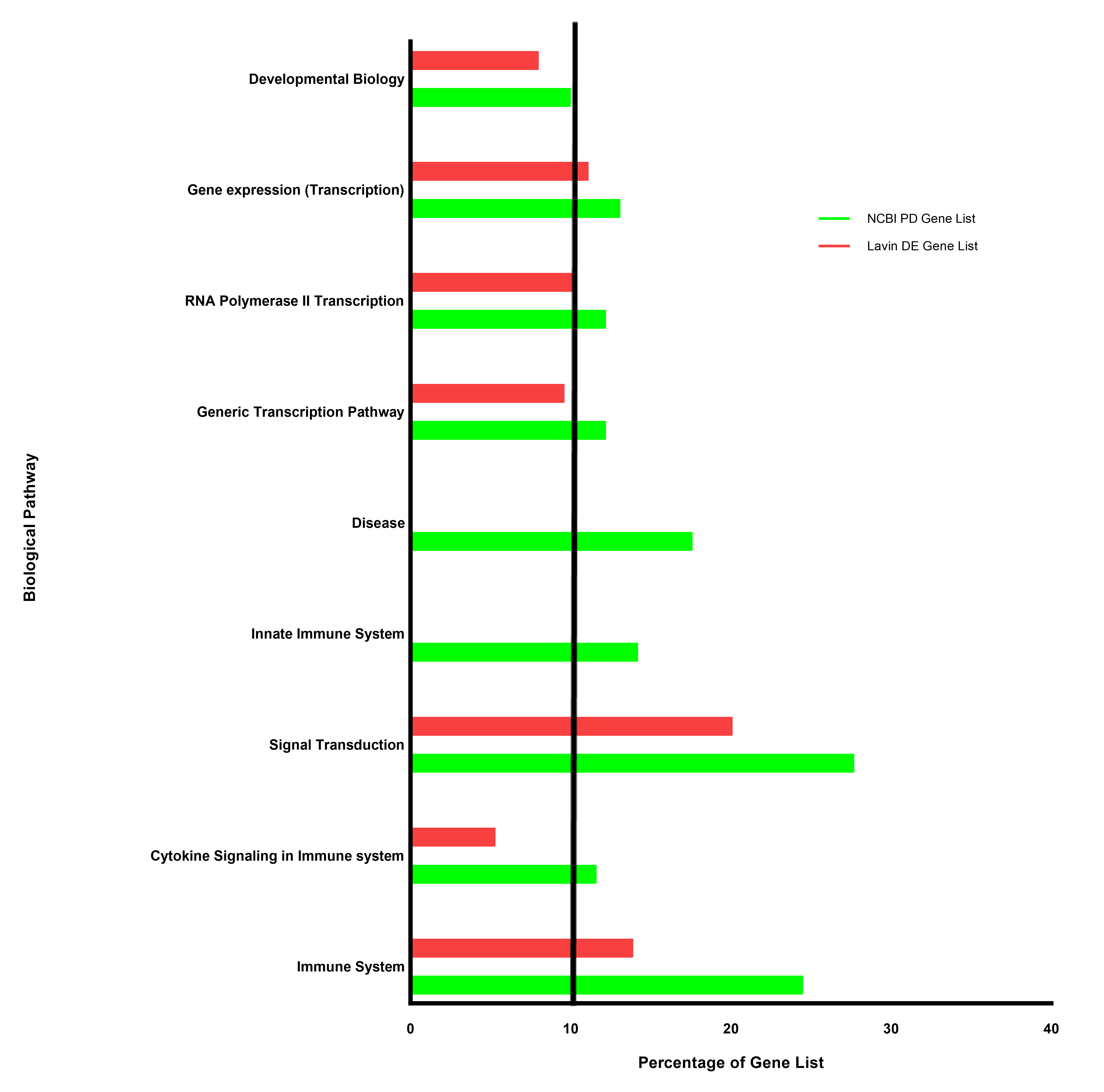

Supplement: S1 Fig — (TIF) [file pone.0287078.s008.tif]

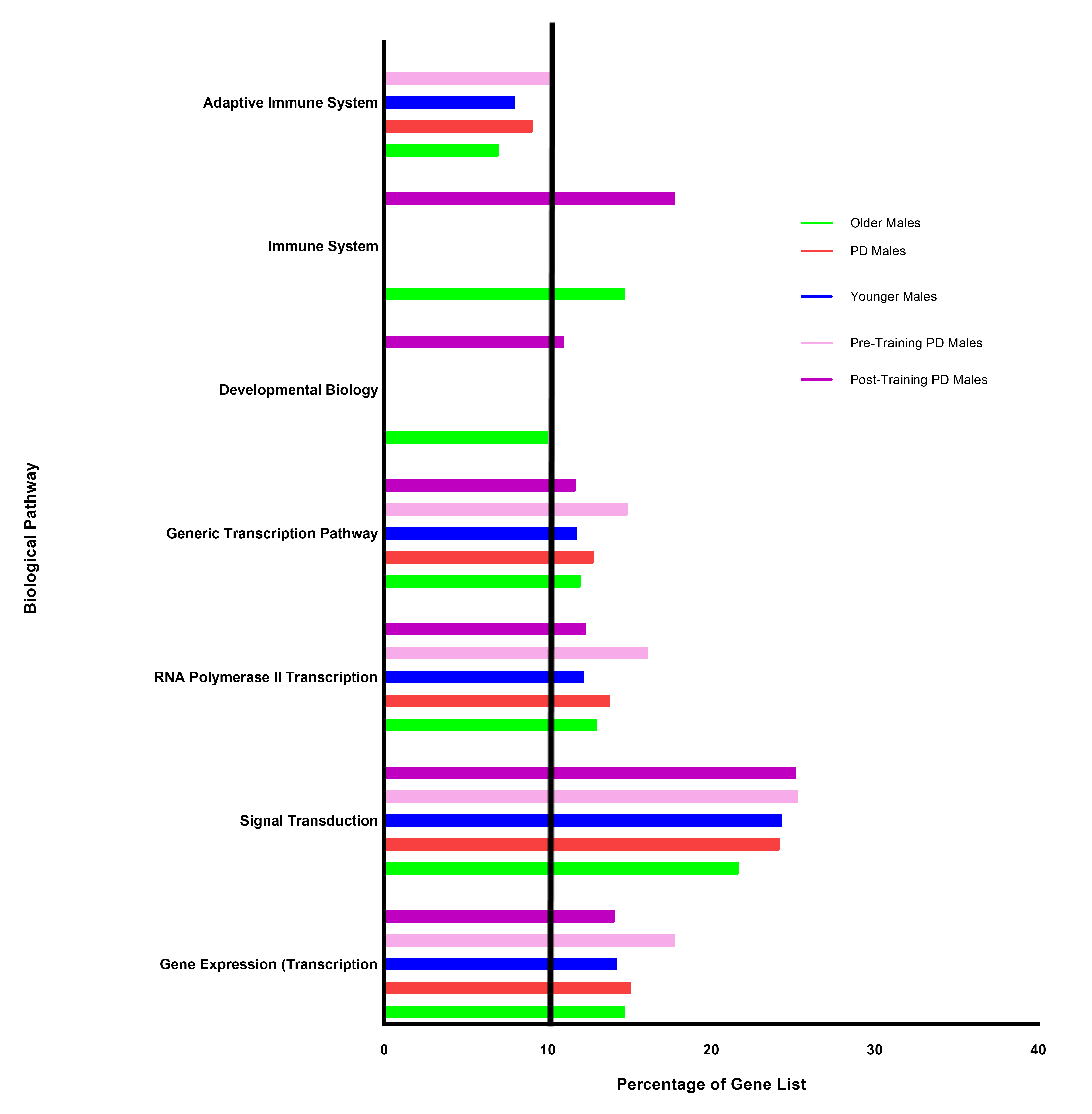

Supplement: S2 Fig — (TIF) [file pone.0287078.s009.tif]

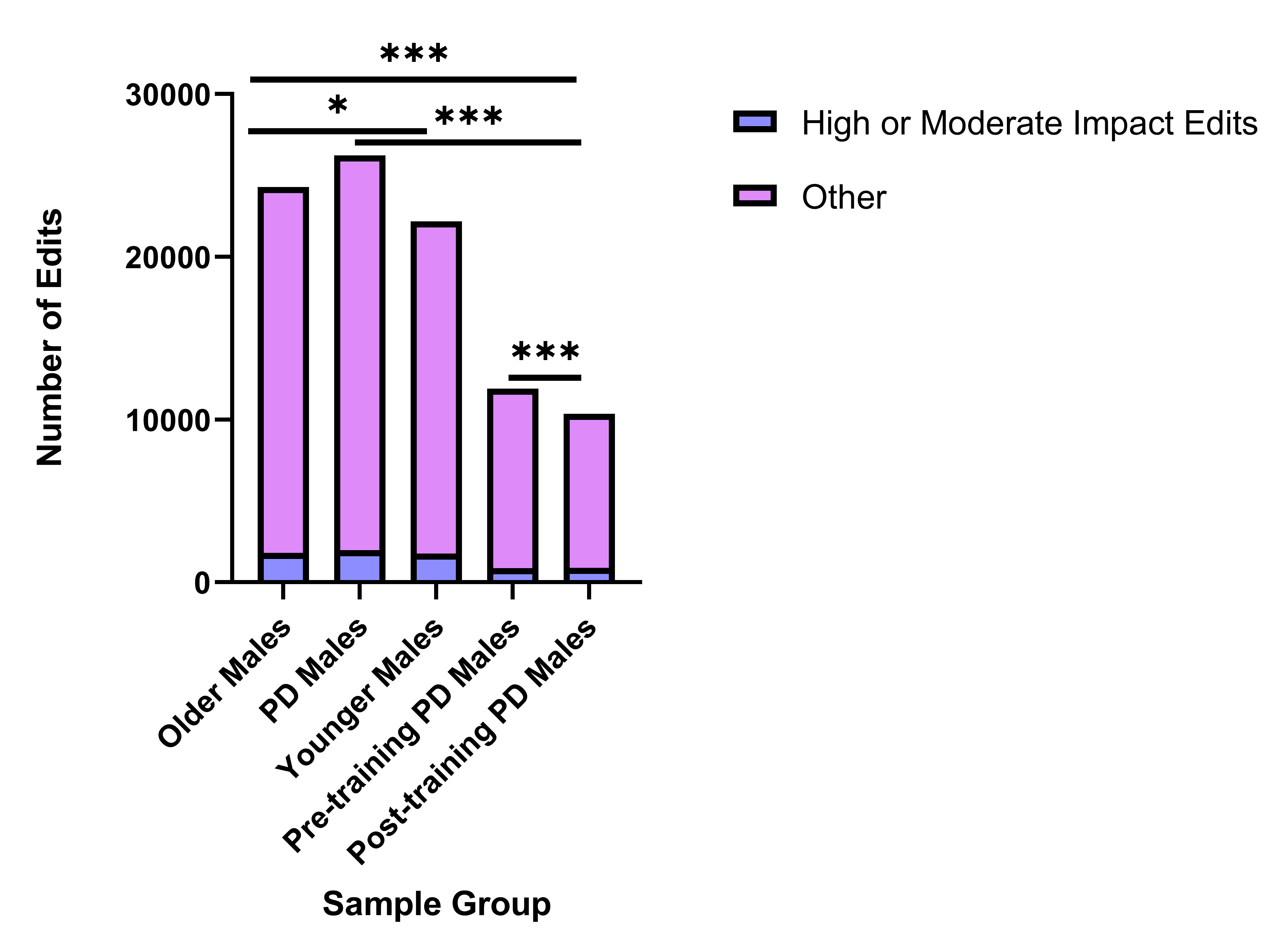

Supplement: S3 Fig — (TIF) [file pone.0287078.s010.tif]

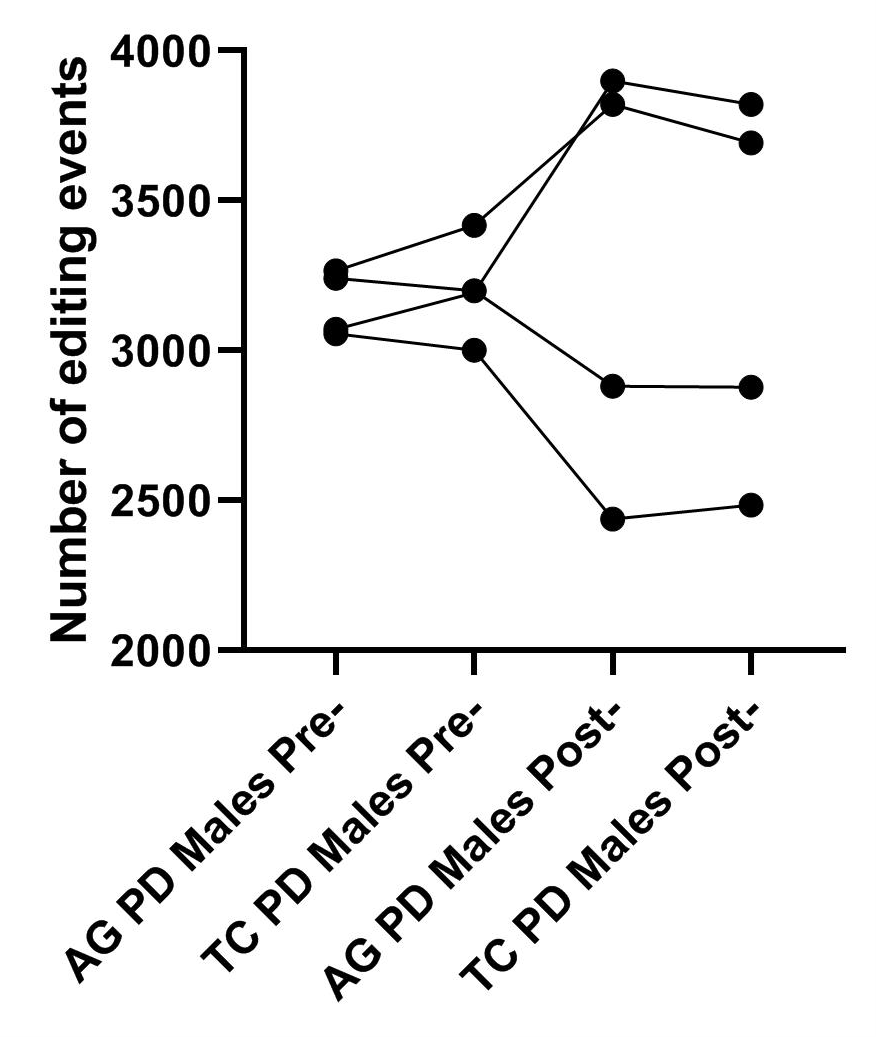

Supplement: S4 Fig — (TIF) [file pone.0287078.s011.tif]

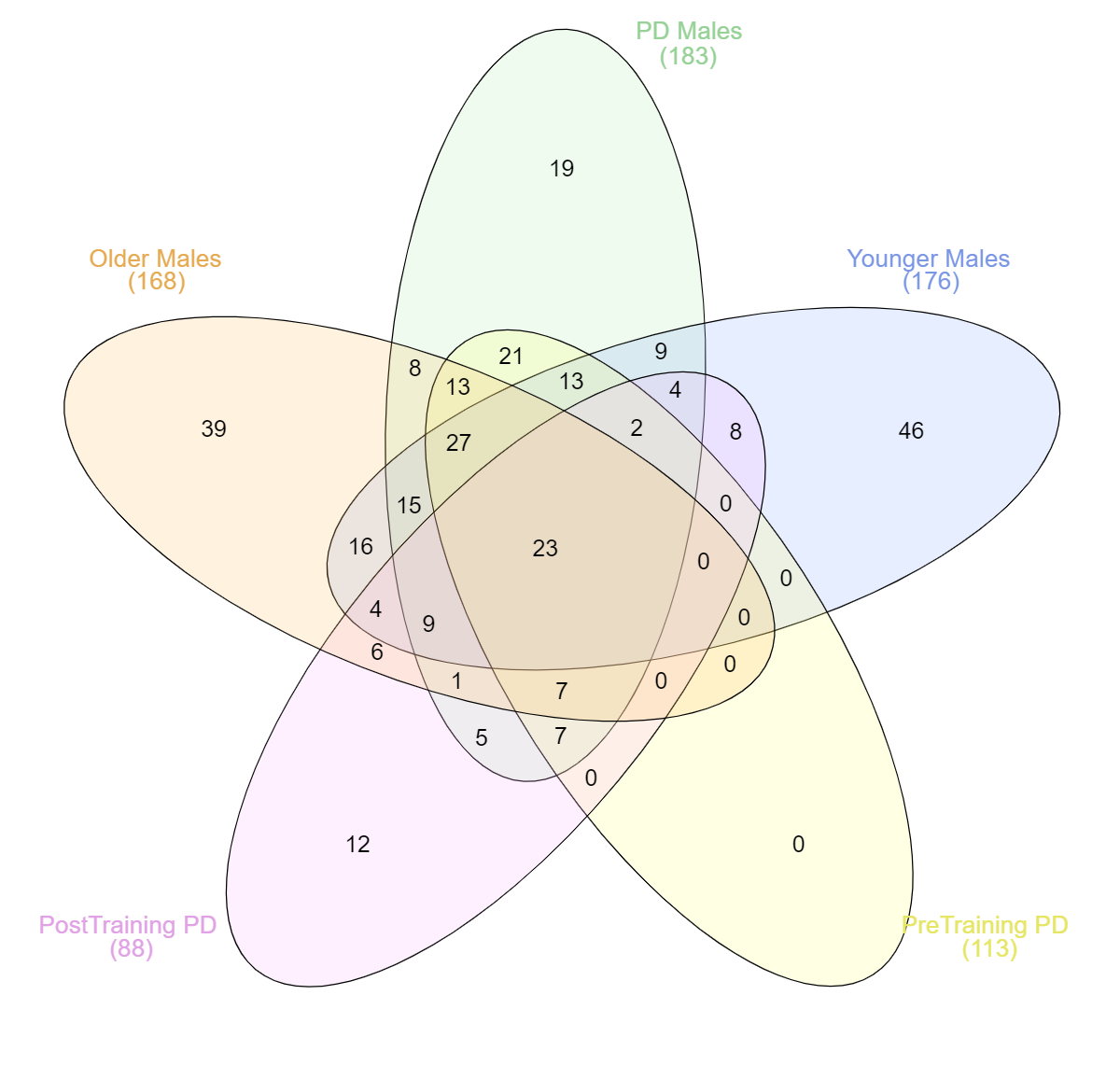

Supplement: S5 Fig — (PNG) [file pone.0287078.s012.png]

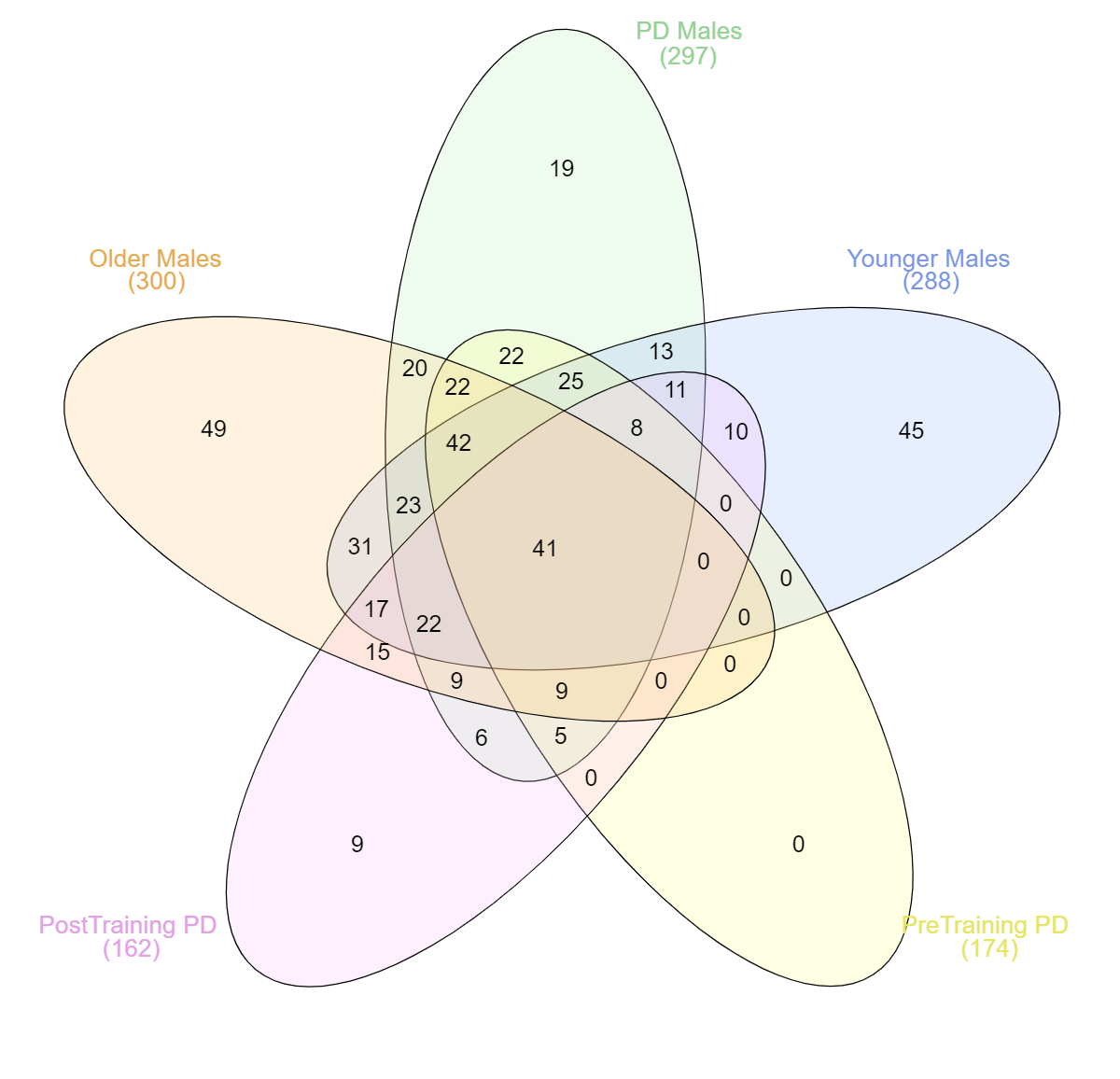

Supplement: S6 Fig — (PNG) [file pone.0287078.s013.png]

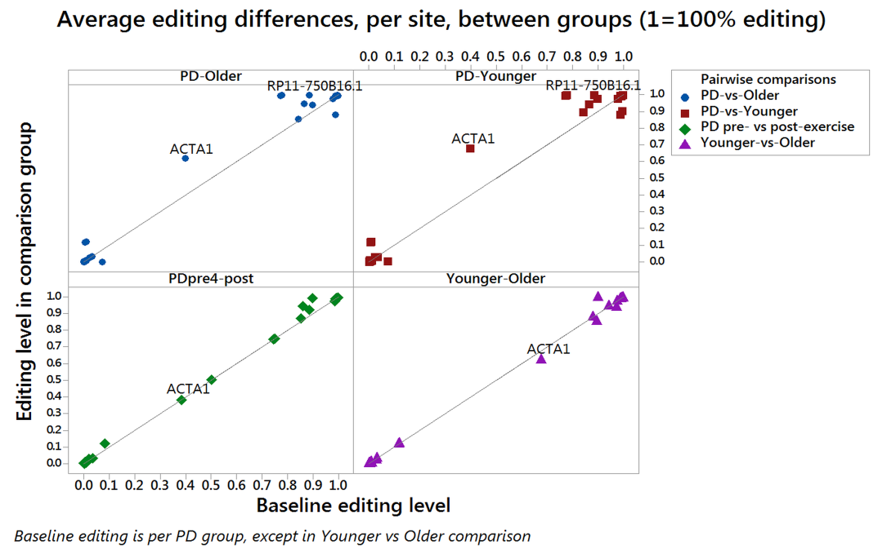

Supplement: S7 Fig — Average editing levels are plotted for each pairwise comparison with each dot representing a single site, for all 55 shared sites. The 45-degree line represents the equal levels of editing in two contrasted groups, for a specific site. For all of these comparisons, except between Younger and Older groups, the X axis (baseline) is per the level of editing within PD group (either the entire 9-patients group, or only the subset of 4 patients that participated in exercises). The further away from the 45-degree line the dot is, the larger the difference in editing between the compared groups, for that specific site. As the scatterplots show, there is somewhat more under-edited sites in PD samples compared to either Younger or Older patients; while differences in editing at specific sites is relatively small in Younger vs Older or PD pre- and post-exercise subset of patients. Examples of such under-edited sites include sites from ACTA1 and RP11–750B16.1 (marked). (TIF) [file pone.0287078.s014.tif]
